# Unique chemical activity in porous YbB_2_C_2_ ceramics with high porosity and high compressive strength

**DOI:** 10.1038/s41598-020-77267-9

**Published:** 2020-11-19

**Authors:** Zhihui Li, Jixin Chen, Hao Zhang, Jinxing Yang, Minmin Hu, Luchao Sun, Zerong Zhang, Yongheng Zhang, Meishuan Li

**Affiliations:** 1 Shenyang National Laboratory for Materials Science, Institute of Metal Research, Chinese Academy of Sciences, Shenyang, 110016 China; 2 School of Materials Science and Engineering, University of Science and Technology of China, Shenyang, 110016 China

**Keywords:** Chemistry, Materials science

## Abstract

High purity layered YbB_2_C_2_ powder is synthesized by a boro/carbothermic reduction method using YbBO_3_, B_4_C and graphite powders as raw materials. Its X-ray diffraction data are presented, and the space group *P*4/*mbm* (No. 127) is confirmed. The lattice parameters are *a* = *b* = 5.3389 Å and *c* = 3.5683 Å, and the atom positions are Yb (0.0000, 0.0000, 0.0000), B (0.3621, 0.8621, 0.5000), and C (0.1606, 0.6606, 0.5000). Porous YbB_2_C_2_ ceramics have a high porosity in the range of 69.89–58.11% and a high compressive strength in the range of 19.49–63.44 MPa. Furthermore, the as-produced porous YbB_2_C_2_ ceramics show unique chemical activity. Porous YbB_2_C_2_ ceramic with a porosity of 69.89% emits so much heat that it can burn a piece of paper when this ceramic is wetted by water. The rate of reaction between the porous YbB_2_C_2_ ceramic and water can be simply controlled by adjusting the porosity. The solid reaction products are YbB_6_, C and an unknown amorphous phase.

## Introduction

REB_2_C_2_ (RE = Y and lanthanides) compounds have a layered crystal structure, which can be described as a stacking of layers, –RE–B_2_C_2_–RE–B_2_C_2_–RE–, along the *z*-direction, with the covalently bonded B-C network consisting of non-regular squares and octagons^1–4^. The structure of REB_2_C_2_ is similar to that of M_*n*+1_AX_*n*_ phases (“MAX phases”, where M is an early transition metal, A is an A-group element, X is C or N, and *n* = 1–3)^5–7^. Two different bonds, namely, the strong metallic-covalent bond of M–X and the weak M–A bond, occur in the MAX phases.

The fabrication methods of REB_2_C_2_ include arc melting^8–12^, the two-step method^13–15^, in situ hot pressing^16,17^ and the boro/carbothermic reduction method derived from the synthesis of transition metal borides^16,18–20^, as summarized in Table 1. In all REB_2_C_2_ ceramics, YbB_2_C_2_ is rather difficult to synthesize^10,14^. Sakai et al.^14^ thought the reason was the high vapor pressure of Yb carbide under high temperature. However, a closer study of Yb shows that there are two other possible reasons. First, the number of 4f valence electrons in Yb is 2, which is different from the 3 of other rare earth metals^21^. Second, the divalent state of 2+ is also reflected in the normalized cell volume *V*_n_ = *V*/*Z* of the compound, which does not follow the usual lanthanide contraction trend^22^. In this work, YbBO_3_ instead of Yb_2_O_3_ was employed to fabricate YbB_2_C_2_ by the boro/carbothermic reduction method. During the process, the possible reaction is shown in Table 1.

**Table 1:** Comparison of different methods for synthesizing REB_2_C_2_.

Methods	Starting materials	Routs or reactions
Arc-melting technique^8–12^	RE, B and C	(1) (RE + B + C)_powder_ → (RE + B + C)_pellet_ (2) (RE + B + C)_pellet_ → (REB_2_C_2_)_single crystal_ (3) (REB_2_C_2_)_single crystal_ → (REB_2_C_2_)_sample_
Two-step	RE_2_O_3_, B and C^13,14^	(1) RE_2_O_3_ + B → REB_6_ (or REB_4_) (2) REB_6_ + RE_2_O_3_ + 9C → 3REB_2_C_2_ + 3CO or 2REB_4_ + RE_2_O_3_ + 11C → 4REB_2_C_2_ + 3CO
	Y, C and B^15^	(1) Y + 2C → YC_2_ (2) YC_2_ + 2B → YB_2_C_2_
In situ hot pressing^16,17^	YH_2_, B_4_C and C	2YH_2_ + B_4_C + 3C → YB_2_C_2_ + 2H_2_
Boro/carbothermic reduction	RE_2_O_3_, B_4_C and C^16,18–20^	RE_2_O_3_ + B_4_C + 6C → 2REB_2_C_2_ + 3CO
	YbBO_3_, B_4_C and C (This work)	4YbBO_3_ + B_4_C + 19C → 4YbB_2_C_2_ + 12CO

To date, most researchers have focused on the magnetic^14,23,24^ or electrical properties^13,15^ of REB_2_C_2_. To our knowledge, only a few researchers have recently used first-principles calculations to study the mechanical properties of REB_2_C_2_ compounds^25,26^. Regarding experimental work, Zhao et al.^16,17^ successfully synthesized high purity bulk YB_2_C_2_ and studied its physical and mechanical properties, which showed excellent damage tolerance, easy machinability, high melting point (2500 °C < *T*_*m*_ < 2600 °C) and excellent high temperature rigidity (the value of Young’s modulus at 1500 °C is still similar to that at room temperature). These features of bulk YB_2_C_2_ ceramic might endow it with great untapped potential for future ultrahigh temperature applications^16,17^. Chen et al.^27^ reported that porous YB_2_C_2_ ceramics fabricated by a high-temperature reaction/partial sintering process possess high porosity (75.26–57.17%), high compressive strength (9.32–34.78 MPa), and anisotropy in the microstructure and mechanical behavior. These features of porous YB_2_C_2_ ceramic render it promising as a thermally insulating lightweight component for transpiration cooling systems^27^.

As the number of 4f valence electrons in Yb is different from those in other rare earth metals, the YbB_2_C_2_ ceramic may possess unique properties. In this work, high purity YbB_2_C_2_ powder was successfully synthesized. The crystal structure and lattice parameters were studied. Then, porous YbB_2_C_2_ ceramics with high porosity and high compressive strength were prepared. In addition, the as-prepared porous YbB_2_C_2_ ceramics were found to possess individual chemical activity in wet environments.

## Experimental

YbB_2_C_2_ powder and porous YbB_2_C_2_ ceramics were synthesized by the boro/carbothermic reduction method with a mixture of YbBO_3_, B_4_C (99%, 200 mesh, Jingangzhuan, China), and graphite (99%, 200 mesh, Tianyuan, China) powders. The routes for fabricating YbB_2_C_2_ powder can be described as follows. First, raw powders with a 4.00:1.28:18.16 molar ratio were milled in a polypropylene jar for 6 h in alcohol to obtain a homogeneous mixture. The powder mixture was then dried in an oven at 60 °C for 24 h. After that, the powder mixture was placed in a graphite crucible heated at a rate of 10 °C/min to 1950 °C and held for 1.0 h under flowing Ar. The route for synthesizing porous YbB_2_C_2_ ceramics can be generalized as follows: at first, the homogeneous mixture powders of YbBO_3_, B_4_C, and graphite (as above) were uniaxially pressed into columnar compacts by applying different pre-pressures (10, 20, 30, 50, 100, 200 and 300 MPa) with a dwell time of 5 min. Then, these green bodies were placed in a graphite crucible and heated at a rate of 8 °C/min to 1980 °C and held for 1.5 h under flowing Ar.

The phase composition was identified using an X-ray diffractometer with Cu Kα radiation (Rigaku D/max-2400, Tokyo, Japan). Rietveld refinement was performed on the XRD results using the GSAS suite with EXPGUI^28,29^. The morphology was investigated by a SUPRA 35 scanning electron microscope (SEM) (LEO, Oberkochen, Germany) equipped with an energy dispersive spectroscopy (EDS) system. Selected area electron diffraction (SAED) was performed using a Tecnai G2 F20 (FEI, Eindhoven, the Netherlands) instrument equipped with a field emission gun. During the process, a perforated carbon/copper net served as a support for the as-prepared powder.

The density of the porous YbB_2_C_2_ ceramics was calculated geometrically, by measuring the volume and weight of five identical samples and averaging the data to ensure accuracy. The porosity (*η*) of porous YbB_2_C_2_ ceramics was determined by Eq. (1), where *ρ* and *ρ*_0_ refer to the sintered density and the theoretical density of YbB_2_C_2_ ceramics, respectively. The compressive strength was measured using a microcomputer control electron universal testing machine (*CMT4204*, Shenzhen SANS testing machine Co., Ltd, Shenzhen, China). The tested samples were rectangular bars with dimensions of 5 mm × 5 mm × 10 mm. The compressive strengths of the as-prepared porous YbB_2_C_2_ ceramics were tested in the directions parallel (σ_//_) and perpendicular (σ_⊥_) to the forming direction. For each group, five samples with the same density were used to obtain the average value. The crosshead speed was 0.5 mm/min

1 $$ \eta = { }\left( {1 - {\raise0.7ex\hbox{$\rho $} \!\mathord{\left/ {\vphantom {\rho {\rho_{0} }}}\right.\kern-\nulldelimiterspace} \!\lower0.7ex\hbox{${\rho_{0} }$}}} \right) \times 100\% $$

## Results and discussion

### XRD analysis and Rietveld refinement of YbB_2_C_2_

**Figure 1:**
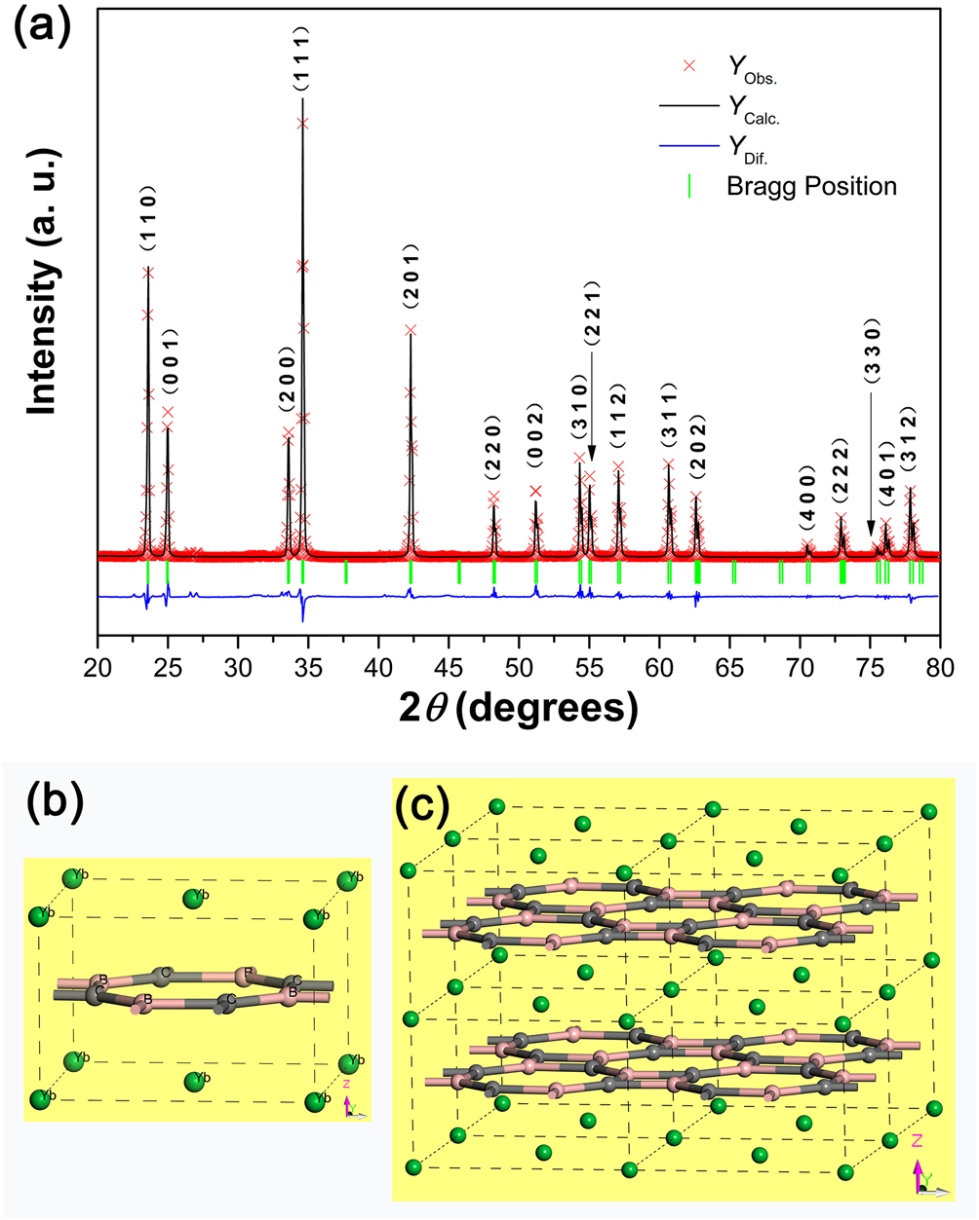
Powder XRD spectra (**a**) of YbB_2_C_2_: observed spectrum (red crosses), Rietveld generated spectrum (black line) and difference between the two (blue line). The green vertical ticks below the pattern represent possible Bragg reflections of YbB_2_C_2_. The crystal structure of YbB_2_C_2_: unit cell (**b**) and 2 × 2 × 2 supercell (**c**).

To date, the crystal structure of REB_2_C_2_ is still controversial. Bauer^2,30^ and Sakai et al.^15^ believed that its space group is *P*-24*c* (No. 112), but an increasing number of researchers’ studies^4,8,10,31,32^ confirmed that its space group is *P*4/*mbm* (No. 127). In this work, the *P*4/*mbm* space group is used as a starting model for refining the YbB_2_C_2_ sample, and the initial values of the cell parameters and atom coordinates come from the isostructural compound YB_2_C_2_^26^. The experimental XRD spectrum (red) and the calculated pattern obtained by Rietveld analysis (black) from 2*θ* = 20° to 80° are shown in Fig. 1a. Only the YbB_2_C_2_ phase is detected, which confirms that high purity YbB_2_C_2_ powder has been successfully synthesized. Considering the difficulty of synthesizing the YbB_2_C_2_ compound^10,14^, the as-fabricated YbB_2_C_2_ powder is remarkable, and the boro/carbothermic reduction method shows more advantages than other methods^8–17^. It should be noted that the difference between the fabricated molar ratio (4.00:1.28:18.16) and theoretical molar ratio (4.00:1.00:19.00) of the raw powders (YbBO_3_, B_4_C and graphite) may come from their different vapor losses under high temperature.

It is well known that the goodness of fit (GOF) for the peak shape and position, structure and background is measured in terms of profile *R* (reliability) factors, and that relatively lower values of *R*_wp_ (the weighted spectrum *R* factor) and *R*_p_ (the spectrum R factor) are considered to indicate good profile refinement^33^. *R*_wp_ and *R*_p_ can be written as Eqs. (2) and (3), where *W*_*i*_, *Y*_*oi*_ and *Y*_*ci*_ refer to the weight factor based on statistics, diffraction intensity from observation and diffraction intensity from calculation, respectively^34^. Obviously, the observed and calculated spectra are in good agreement, and low values of *R*_wp_ (10.37%) and *R*_p_ (7.52%) are obtained

2 $$ R_{wp} = \left[ {{{W_{i} \left( {Y_{oi} - Y_{ci} } \right)^{2} } \mathord{\left/ {\vphantom {{W_{i} \left( {Y_{oi} - Y_{ci} } \right)^{2} } {W_{i} Y_{oi}^{2} }}} \right. \kern-\nulldelimiterspace} {W_{i} Y_{oi}^{2} }}} \right]^{{{\raise0.7ex\hbox{$1$} \!\mathord{\left/ {\vphantom {1 2}}\right.\kern-\nulldelimiterspace} \!\lower0.7ex\hbox{$2$}}}} $$

3 $${R}_{p}=\sum \left|{Y}_{oi}-{Y}_{ci}\right|/\sum {Y}_{oi}$$

The lattice parameters of YbB_2_C_2_ calculated from the refinement are *a* = *b* = 5.3389 Å and *c* = 3.5683 Å, respectively, and the atom positions are Yb (0.0000, 0.0000, 0.0000), B (0.3621, 0.8621, 0.5000), and C (0.1606, 0.6606, 0.5000). The crystal structure of YbB_2_C_2_ can be seen in Fig. 1b,c. The results are in accordance with the cell parameters and (or) atom positions of the known REB_2_C_2_ compound^4,11,26,32^. The detailed XRD results of the structural refinements are grouped in Table 2. In addition, the theoretical density of the YbB_2_C_2_ ceramic calculated from the refinement is 7.05 g/cm^3^.

**Table 2:** Reflections, 2*θ* positions, d (interplanar spacing) and intensity data of YbB_2_C_2_ powder from observation (Obs.) and calculation (Calc.).

Reflection (*hkl*)	2*θ*_Obs_ (°)	2*θ*_Calc_ (°)	d_Obs_ (Å)	d_Calc_ (Å)	I/I_0Obs_ (%)	I/I_0Calc_ (%)
110	23.60	23.55	3.775	3.785	65.74	63.50
001	24.96	24.93	3.568	3.593	30.46	28.28
200	33.60	33.54	2.669	2.677	29.22	26.48
111	34.60	34.56	2.593	2.606	100.00	100.00
201	42.29	42.24	2.138	2.146	52.63	48.91
220	48.22	48.17	1.888	1.893	14.57	11.78
002	51.21	51.16	1.784	1.797	15.76	12.44
310	54.32	54.29	1.688	1.693	23.38	21.03
221	55.04	54.99	1.669	1.674	19.20	16.17
112	57.07	57.05	1.613	1.623	21.36	19.26
311	60.65	60.63	1.526	1.531	21.91	20.55
202	62.61	62.57	1.483	1.492	14.48	13.63
400	70.53	70.49	1.335	1.338	3.59	3.20
222	72.92	72.89	1.297	1.303	9.62	9.31
330	75.51	75.48	1.258	1.262	2.93	2.78
401	76.11	76.07	1.250	1.254	8.41	7.86
312	77.86	77.83	1.226	1.232	15.78	15.64
411	78.50	78.52	1.217	1.221	0.85	0.80

### Microstructure of the YbB_2_C_2_ powder

**Figure 2:**
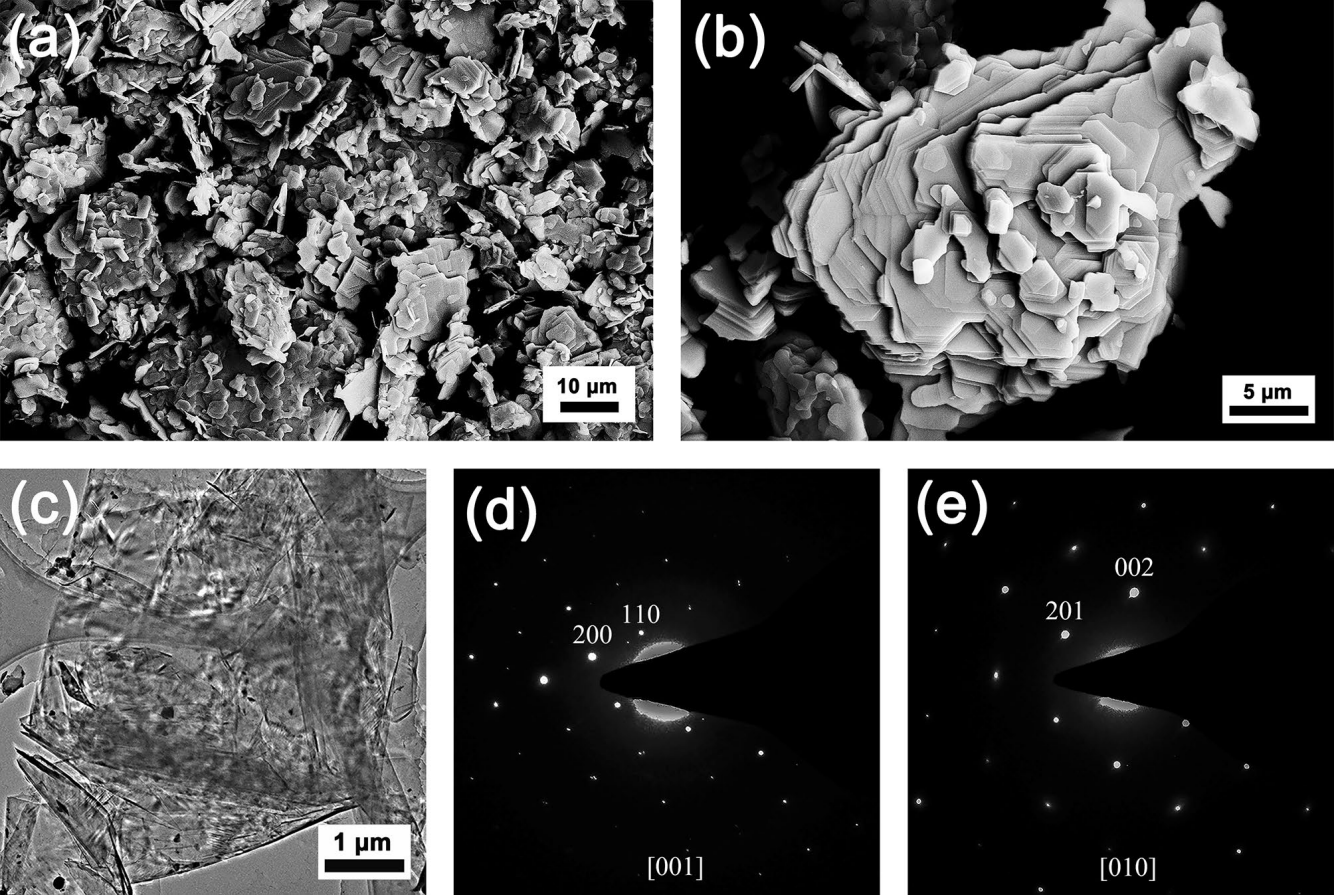
SEM images of the YbB_2_C_2_ powder: low magnification (**a**) and high magnification (**b**). TEM image of the YbB_2_C_2_ powder (**c**). SAED patterns corresponding to the YbB_2_C_2_: [001] (**d**) and [010] (**e**) zone axes.

The morphology of the YbB_2_C_2_ powder observed by SEM is shown in Fig. 2a,b. The YbB_2_C_2_ powder consists of typical laminated plates with a size of approximately several microns in thickness and tens of microns in length and width. The laminated plates of YbB_2_C_2_ reflect the alternate arrangement of the B–C networks and Yb sheets, as shown in Fig. 1c. To clearly observe the morphology of the YbB_2_C_2_ powder, TEM was used as well. Figure 2c shows the bright field image (BFI) of YbB_2_C_2_. The as-prepared YbB_2_C_2_ possesses a typical laminar microstructure. Figure 2d,e show the diffraction results along the [001] and [010] zone axes. All maxima can be indexed on the basis of a tetragonal cell, with parameters *a* = 5.2835 Å and *c* = 3.4920 Å; both values are in good agreement with those measured results from the XRD spectra. In addition, Fig. 2d,e and Table 2 show that the reciprocal space spots were not extinct as long as they met “0*kl*: *k* = 2*n*, *h*00*: h* = 2*n* or *hkl*: *h* + *k* = 2*n*”. According to the *International Tables of Crystallography*^35^, the reflection conditions are compatible with the space group *P*4/*mbm* (No. 127), which confirms the previous choice for Rietveld refinement.

### Porous YbB_2_C_2_ ceramics

**Figure 3:**
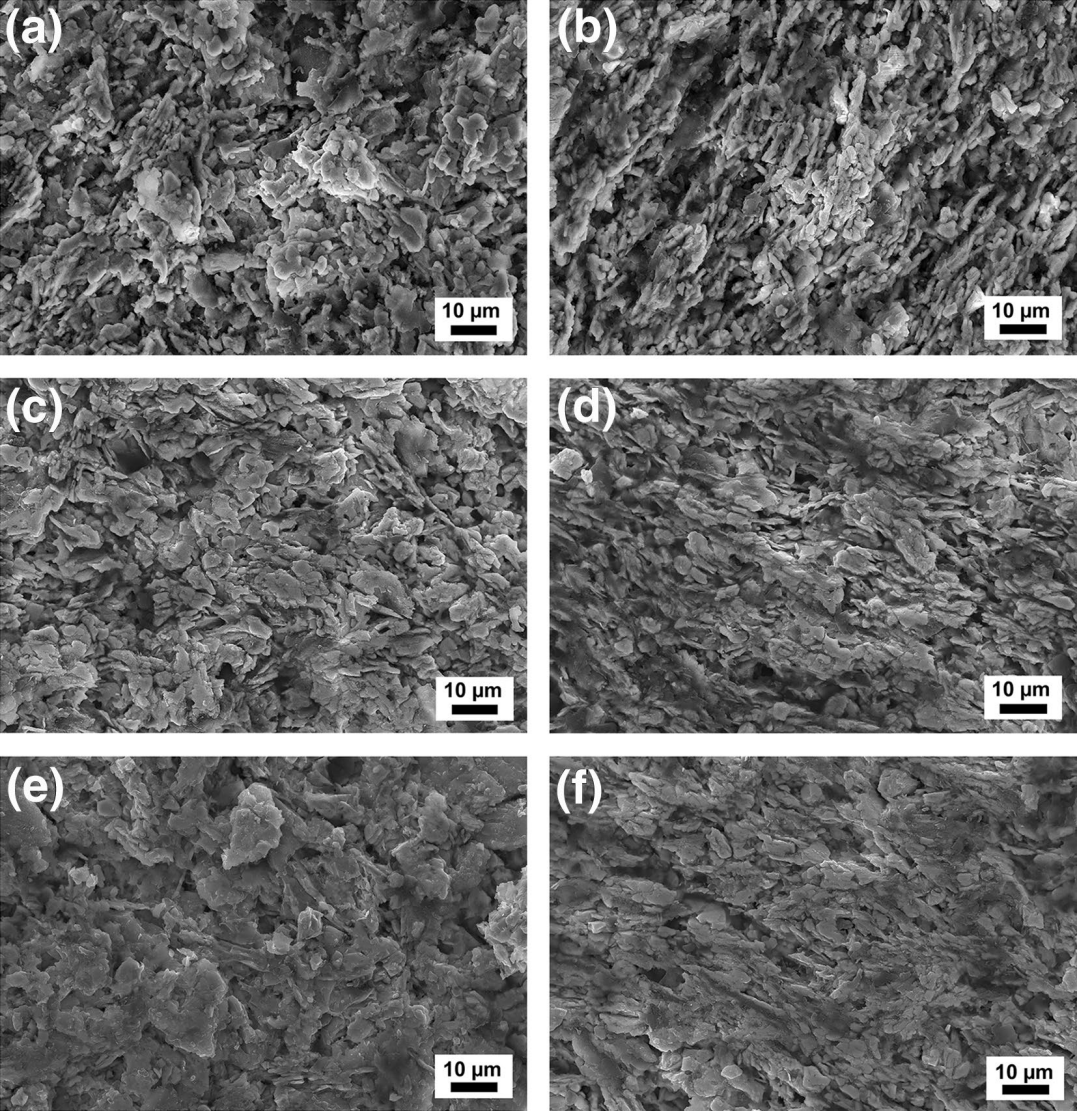
SEM images of the as-prepared porous YbB_2_C_2_ samples with different porosities. Top surface perpendicular to the forming direction (**a**) and cross section parallel to the forming direction (**b**) of the as-prepared sample (*η* = 69.89%). Top surface (**c**) and cross section (**d**) of the sample (*η* = 61.58%). Top surface (**e**) and cross section (**f**) of the sample (*η* = 58.11%).

Advanced porous ceramics (also called ceramic foams) usually have low density, high porosity, large specific surface area, thermal shock resistance, corrosion and wear resistance, and high chemical stability^36–38^. SEM images of the as-prepared porous YbB_2_C_2_ samples with porosities of 69.89%, 61.58% and 58.11% are shown in Fig. 3. The microstructure can be described as an incompact stacking of uniform lamellae with a size of approximately one or two microns in thickness and tens of microns in length and width. It should be noted that the microstructure of porous YbB_2_C_2_ is slightly different from that of porous YB_2_C_2_. This difference in microstructure comes from the different kinds of raw materials employed. For porous YB_2_C_2_ ceramics, Y_2_O_3_, BN and C were used as raw materials, and small grains and large lamellar grains were obtained due to the in situ reaction process and inheritance from graphite, respectively^27^. In the porous YbB_2_C_2_ ceramics, the lamellas are oriented to a certain degree, more or less, in the plane perpendicular to the forming direction; this may be due to the raw material of graphite, which has a typical lamellar structure. The anisotropic microstructure of the porous YbB_2_C_2_ becomes more obvious with decreasing porosity.

**Figure 4:**
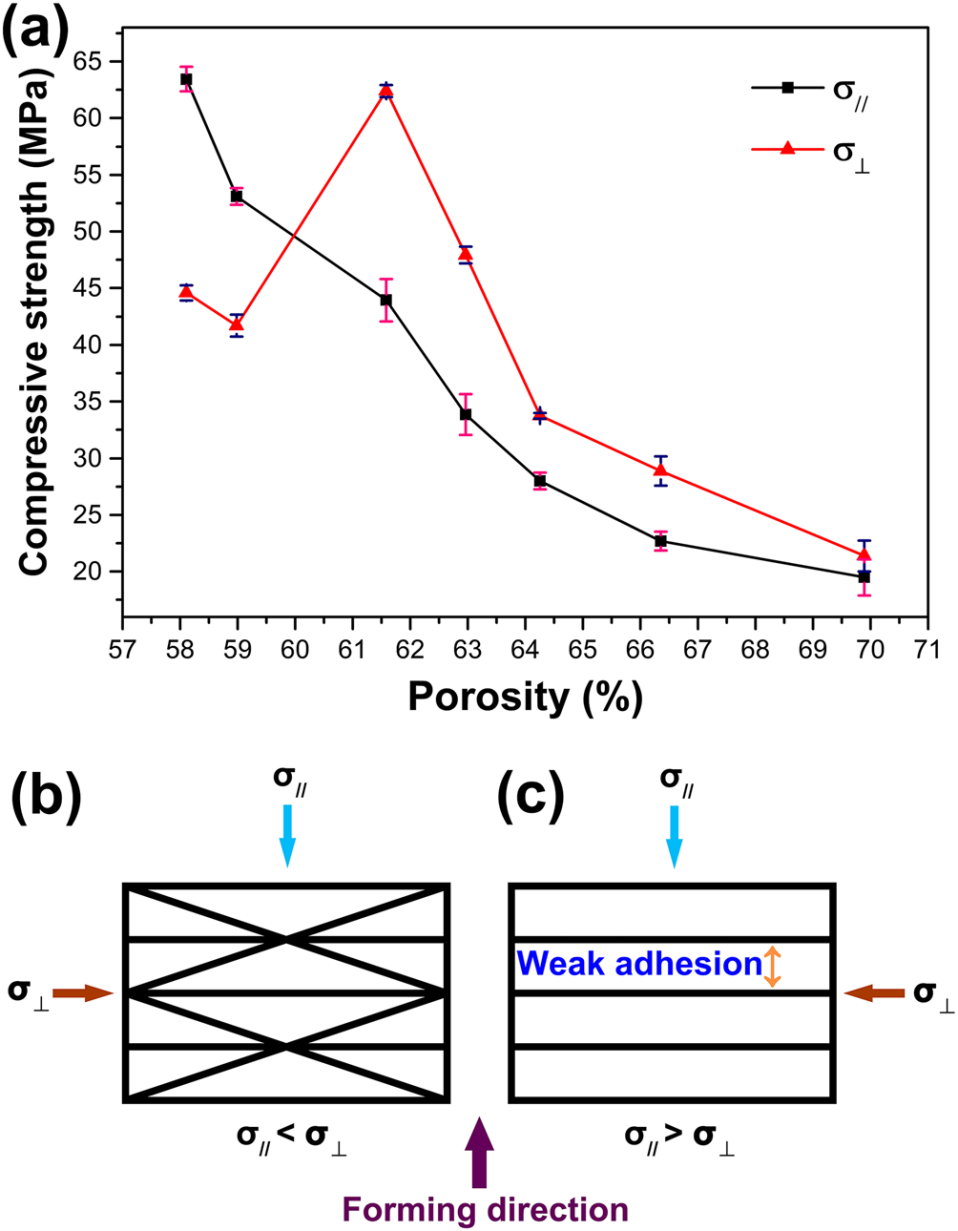
Compressive strengths vs porosities of porous YbB_2_C_2_ samples (**a**). Model of the influence of the anisotropic microstructure of porous YbB_2_C_2_ samples on their compressive strengths: the lamellas of the porous YbB_2_C_2_ samples are oriented to a small degree in the plane perpendicular to the forming direction (**b**) and the lamellas of the porous YbB_2_C_2_ samples are oriented ideally in the plane perpendicular to the forming direction (**c**).

For porous ceramics, the compressive strength is an important parameter. To confirm the anisotropic mechanical properties of porous YbB_2_C_2_ samples, a compression test was carried out in two directions: σ_//_ (the loading direction parallel to the forming direction) and σ_⊥_ (the loading direction perpendicular to the forming direction), as shown in Fig. 4a and Table 3. The σ_//_ strengths of the as-prepared porous YbB_2_C_2_ samples with porosities of 69.89–58.11% are in the range of 19.49–63.44 MPa. It is obvious that the σ_//_ strength decreases with increasing porosity. Different from the trend of the σ_//_ strength, the maximum σ_⊥_ strength occurs at a porosity of 61.58% (62.40 MPa). The σ_⊥_ strengths are higher than the corresponding σ_//_ strengths for samples with porosities of 69.89–61.58%, while the former are lower than the latter for samples with porosities of 58.98–58.11%. Two factors determine the σ_⊥_ strengths, namely, the porosity and anisotropic microstructure. With increasing porosity, the σ_⊥_ strength decreases as well, which is similar to the trend of the σ_//_ strength. The anisotropic microstructure, i.e., the YbB_2_C_2_ lamellas are oriented in the plane perpendicular to the forming direction has an adverse effect on the σ_⊥_ strength. This may be due to the weak adhesion between the YbB_2_C_2_ laminates. The influence of the anisotropic microstructure of the porous YbB_2_C_2_ samples on their compressive strengths can be understood by a model, as shown in Fig. 4b,c. On one hand, when lamellas of porous YbB_2_C_2_ samples are oriented to a small degree in the plane perpendicular to the forming direction (Fig. 4b), some lamellas act as diagonal bracings that enhance the σ_⊥_ strength (samples with porosity of 69.89–61.58%, σ_//_ < σ_⊥_). On the other hand, when the lamellas of the porous YbB_2_C_2_ samples are oriented ideally in the plane perpendicular to the forming direction (Fig. 4c), the oriented lamellas easily disintegrate because of the weak adhesion between them, which weakens the σ_⊥_ strength (samples with porosities of 58.98–58.11%, σ_//_ > σ_⊥_). For the porous YbB_2_C_2_ sample with a porosity of 61.58%, the porosity is not high enough and the lamellas are oriented to an appropriate degree. These two factors work together and ultimately enhance the σ_⊥_ strength (a maximum of 62.40 MPa). According to Chen et al.^27^, porous YB_2_C_2_ ceramics also exhibit anisotropy in their mechanical properties: samples with porosities of 75.26–57.17% possess σ_//_ strengths of 9.32–34.78 MPa, samples with porosities of 72.40–54.02% possess σ_⊥_ strengths of 17.47–98.57 MPa. However, the mechanism of the anisotropic properties in porous YbB_2_C_2_ ceramics is different from that in porous YB_2_C_2_ ceramics. For the latter, the mechanism can be described as follows: the failure of σ_//_ is caused by accumulated damage^27,39^, and the failure of σ_⊥_ is caused by permanent damage^27,40^.

**Table 3:** Pre-pressure, sintered density, porosity and compressive strength of the as-prepared porous YbB_2_C_2_ samples.

Sample	Pre-pressure (MPa)	Sintered density (g/cm^3^)	Porosity (%)	Compressive strength (MPa)	
				σ_//_	σ_⊥_
1	10	2.12	69.89	19.49	21.38
2	20	2.38	66.35	22.70	28.88
3	30	2.51	64.26	28.00	33.75
4	50	2.61	62.96	33.86	47.93
5	100	2.71	61.58	43.95	62.40
6	200	2.89	58.98	53.10	41.70
7	300	2.95	58.11	63.44	44.59

**Figure 5:**
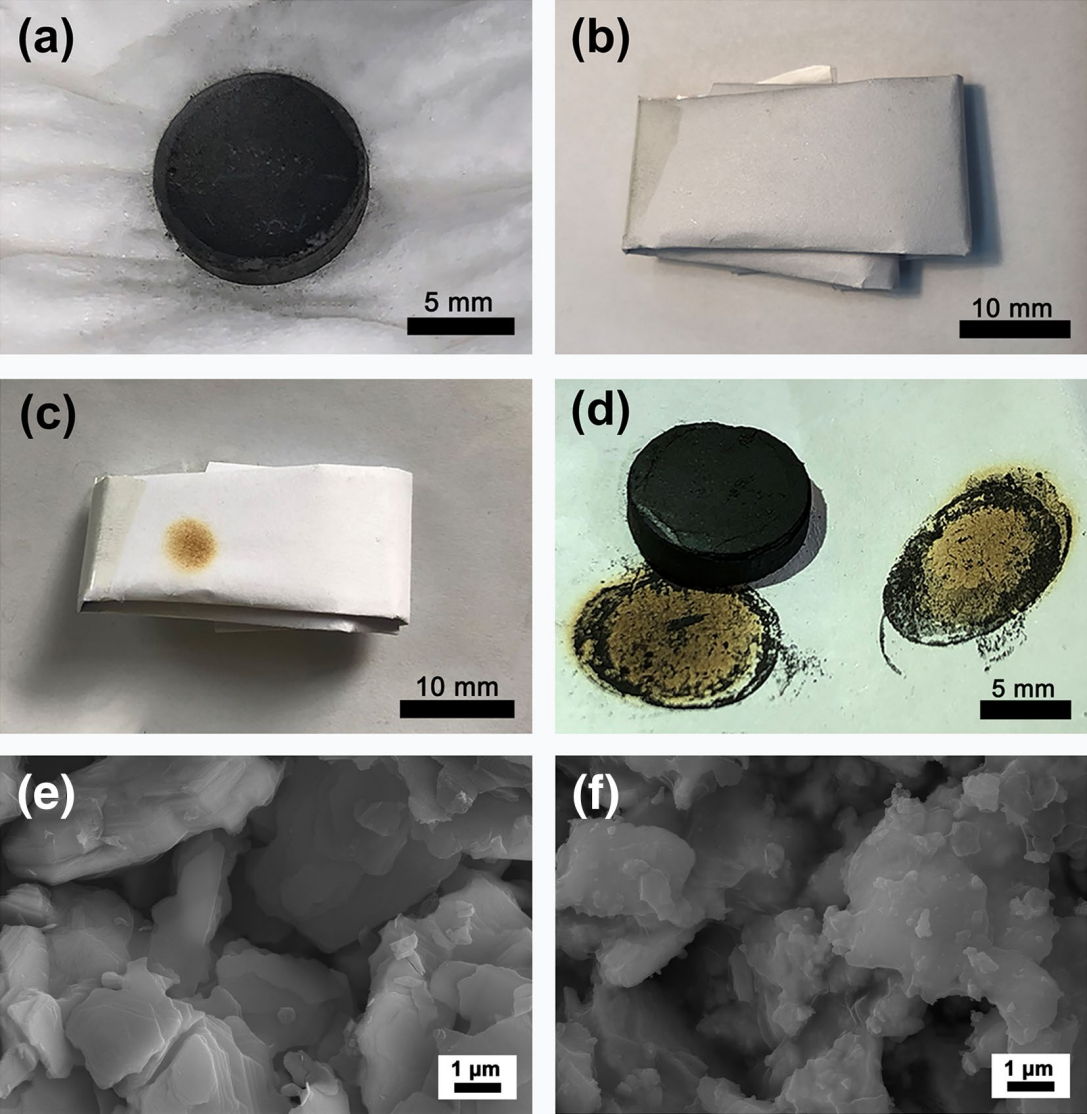
Photographs of the as-prepared porous YbB_2_C_2_ sample (*η* = 69.89%) and its reaction with water (**a**–**d**). SEM micrograph of the as-prepared porous YbB_2_C_2_ sample (*η* = 69.89%) (**e**). SEM micrograph of the solid reaction products of the YbB_2_C_2_ sample (*η* = 69.89%) with water for 3 h (**f**).

**Figure 6:**
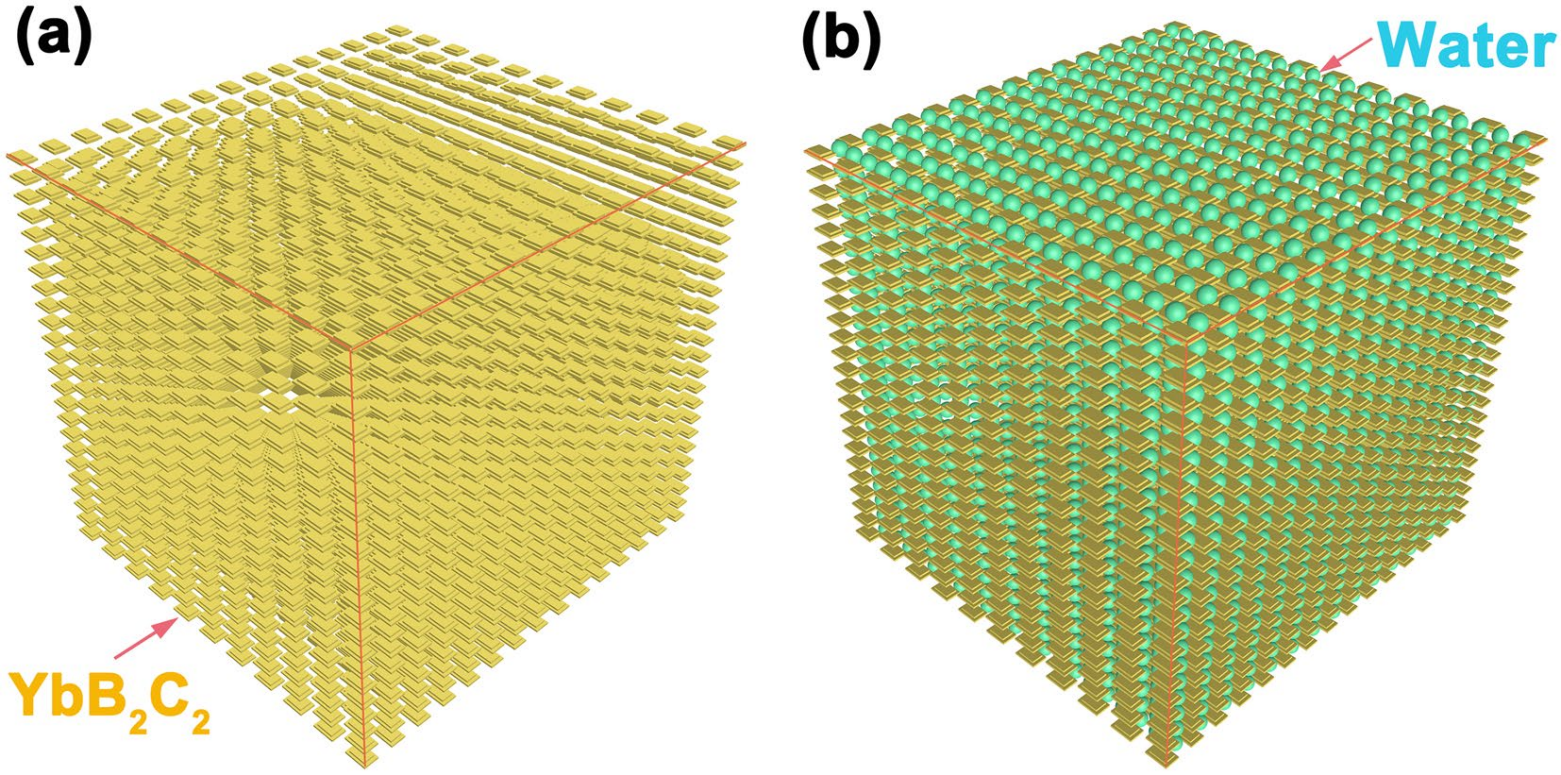
Sponge-like structure of the porous YbB_2_C_2_ sample (**a**). The structure of the water-absorbed YbB_2_C_2_ sample (**b**).

In addition, the as-prepared porous YbB_2_C_2_ ceramics were found to produce a large amount of heat when they were wetted by water. One typical example is the reaction between a porous YbB_2_C_2_ sample (Φ10 × 3 mm) with a porosity of 69.89% and water, as shown in Fig. 5a–d. The sample was first immersed in deionized water for 1 min (Fig. 5a), then the soaked sample was wrapped by a piece of A4 paper (Fig. 5b), and the paper was completely burned up after about approximately 3 h (Fig. 5c,d). It is clear that a large amount of heat was emitted during the reaction process. This result implies that porous YbB_2_C_2_ ceramics possess individual chemical activity in wet environments, which is different from the chemical inertness of other porous ceramics^37,38^. It was also found that the reaction between porous YbB_2_C_2_ ceramic and water became slow and non-obvious with decreasing porosity. For example, when the porous YbB_2_C_2_ ceramic’s porosity was reduced to 58.11%, the paper could not be burned up by reaction heat. Obviously, the chemical activity of the porous YbB_2_C_2_ ceramics can be controlled by adjusting their porosity, which can be understood by a sponge model (Fig. 6). During the reaction process, the porous YbB_2_C_2_ ceramic acts as a sponge that can easily soak up water at the beginning. The soaked water can keep the reaction going, while a large amount of heat begins to accumulate. Finally, the wrapped paper was burned up by the accumulated heat.

**Figure 7:**
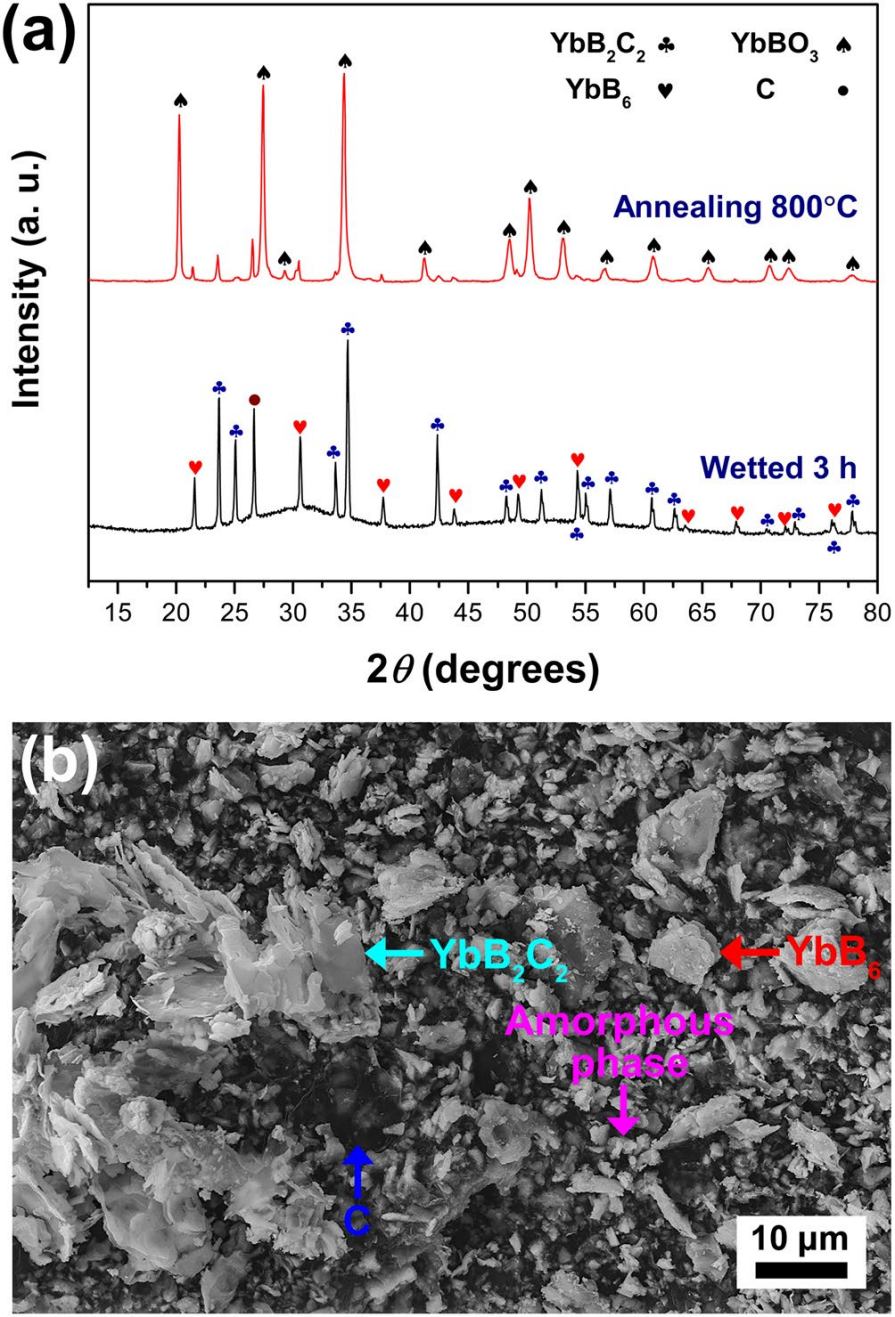
XRD spectrum (**a**) and SEM images (**b**) of solid reaction products of the as-prepared porous YbB_2_C_2_ sample (*η* = 69.89%) with water for 3 h.

Figure 5e,f show the microstructures of the as-prepared porous YbB_2_C_2_ sample and its solid reaction products with water for 3 h. The laminated plates of the porous YbB_2_C_2_ sample are smooth (Fig. 5e), while the plates of its products with water are rough and look like floccules (Fig. 5f). The XRD spectrum of the solid reaction products of the as-prepared porous YbB_2_C_2_ sample with water for 3 h is shown in Fig. 7a. Obviously, apart from the residual YbB_2_C_2_, the solid products consist of YbB_6_, C and an unknown amorphous phase. The amorphous phase can be converted into YbBO_3_ by annealing (800 °C for 1 h under argon flow). Figure 7b shows the morphologies of the solid products. According to the EDS analysis, the lamellas are residual YbB_2_C_2_, the small particles are amorphous phases, the white plates are YbB_6_, and the black plates are graphite.

## Conclusions

High purity YbB_2_C_2_ powder was successfully synthesized by the boro/carbothermic reduction method. The space group *P*4/*mbm* (No. 127) of YbB_2_C_2_ was confirmed. Its X-ray diffraction data, lattice parameters and atom positions were obtained as well. The as-prepared YbB_2_C_2_ powder possesses the typical feature of a layered microstructure.The porous YbB_2_C_2_ ceramics possess a high porosity of 69.89–58.11% and a high compressive strength of 19.49–63.44 MPa, and show unique chemical activity in wet environments. The reaction between the porous YbB_2_C_2_ ceramic and water can be controlled by changing the porosity. The solid reaction products of the as-prepared porous YbB_2_C_2_ sample with water consist of YbB_6_, C and an unknown amorphous phase. The amorphous phase can be converted into YbBO_3_ by annealing.
